# High CLEC-2 expression associates with unfavorable postoperative prognosis of patients with clear cell renal cell carcinoma

**DOI:** 10.18632/oncotarget.11606

**Published:** 2016-08-25

**Authors:** Ying Xiong, Li Liu, Yu Xia, Jiajun Wang, Wei Xi, Qi Bai, Yang Qu, Qilai Long, Jiejie Xu, Jianming Guo

**Affiliations:** ^1^ Department of Urology, Zhongshan Hospital, Fudan University, Shanghai 200032, China; ^2^ Department of Biochemistry and Molecular Biology, School of Basic Medical Sciences, Fudan University, Shanghai 200032, China

**Keywords:** c-type lectin-like receptor 2, clear cell renal cell carcinoma, prognostic factor, overall survival, recurrence-free survival

## Abstract

We enrolled a total of 277 patients who received nephrectomy due to clear cell renal cell carcinoma (ccRCC) in Zhongshan Hospital from Jan 2005 to Jun 2007. Immunohistochemistry was performed to evaluate the impact of CLEC-2 positive cell infiltration on the overall survival (OS) and recurrence-free survival (RFS) of patients with ccRCC. Kaplan-Meier analysis showed that high CLEC-2 positive cell infiltration in tumor tissue indicated poorer OS and RFS (OS, *p* < 0.001; RFS, *p* = 0.002). High CLEC-2 positive cell infiltration is also an independent risk factor for OS and RFS in multivariate analyses (OS, *p* = 0.004; RFS, *p* = 0.009). CLEC-2 positive cell infiltration could also stratify ccRCC patients' survival with University of California Integrated Staging System (UISS) stratum in the mediate-risk and high-risk groups. We constructed two nomograms incorporating parameters derived from multivariate analyses to predict patients' OS and RFS (OS, c-index 0.813; RFS, c-index 0.716). In conclusion, high CLEC-2 positive cell infiltration in ccRCC is an independent adverse prognostic factor for patients, and established nomograms based on this information could help predict ccRCC patients' OS and RFS.

## INTRODUCTION

Renal cell carcinoma (RCC) is the most common malignant cancer in the kidney, accounting for 2% to 3% of all adult malignancies [1], and clear cell renal cell carcinoma (ccRCC) is the most common histological subtype, responsible for most deaths. There are approximately 16.6 women and 37.7 men diagnosed with RCC per 100,000 people every year in China [2]. The natural history of renal cell carcinoma is very complicated and about 20-40% patients would develop recurrences or metastasis even after undergoing curative nephrectomy [3]. Currently, TNM stage, Fuhrman grade and several integrated models like University of California Integrated Staging System (UISS), and Mayo Clinic stage, size, grade and necrosis (SSIGN) score are being used to predict the clinical outcome of RCC. However, these models may not be enough due to the genetic complexity and heterogeneity of the disease [4]. A more accurate prediction model is needed and combining some important molecular biomarkers with current models is probably a new and effective way.

The c-type lectin-like receptor 2 (CLEC-2) known as an emerging pattern recognition receptors for the activation of innate immunity, is a type II membrane protein with a c-type lectin like domain and a single hemITAM motif. CLEC-2 was first identified in a bioinformatic screen in search of c-type lectin receptors and CLEC-2 mRNA was found in the liver and myeloid cells including monocytes, dendritic cells, NK cells, and granulocytes [5]. CLEC-2 signaling modulates toll-like receptor agonists and promotes induction of IL-10 [6]. Later on after systematically analyzing, CLEC-2 was recognized as a platelet activating receptor for the snake venom toxin rhodocytin inducing platelet aggregation. CLEC-2 also possesses an endogenous ligand, the mucin-like glycoprotein podoplanin found on lymphatic endothelium, stromal of secondary lymphoid organs and some cancer cells [7]. Ligation of CLEC-2 with podoplanin elicits strong platelet activation, and it is identified that platelet activation is known to promote tumor metastasis, which may be triggered by podoplanin up-regulation [8]. All these evidence suggests a potential role of CLEC-2 in cancer immunomodulation and metastasis.

However, no researchers have assessed the correlation between clec-2 positive cell infiltration and clinical outcomes of ccRCC patients before. We wondered whether CLEC-2 could become a potential prognostic marker for patients with RCC. Thus we looked into CLEC-2 positive cell infiltration in a large set of clear cell RCC patients by means of immunohistochemistry. The impact of CLEC-2 positive cell infiltration on patients' overall survival (OS) and recurrence-free survival (RFS) were analyzed.

## RESULTS

### Associations between CLEC-2 positive cell infiltration and clinicopathological characteristics

**Figure 1:**
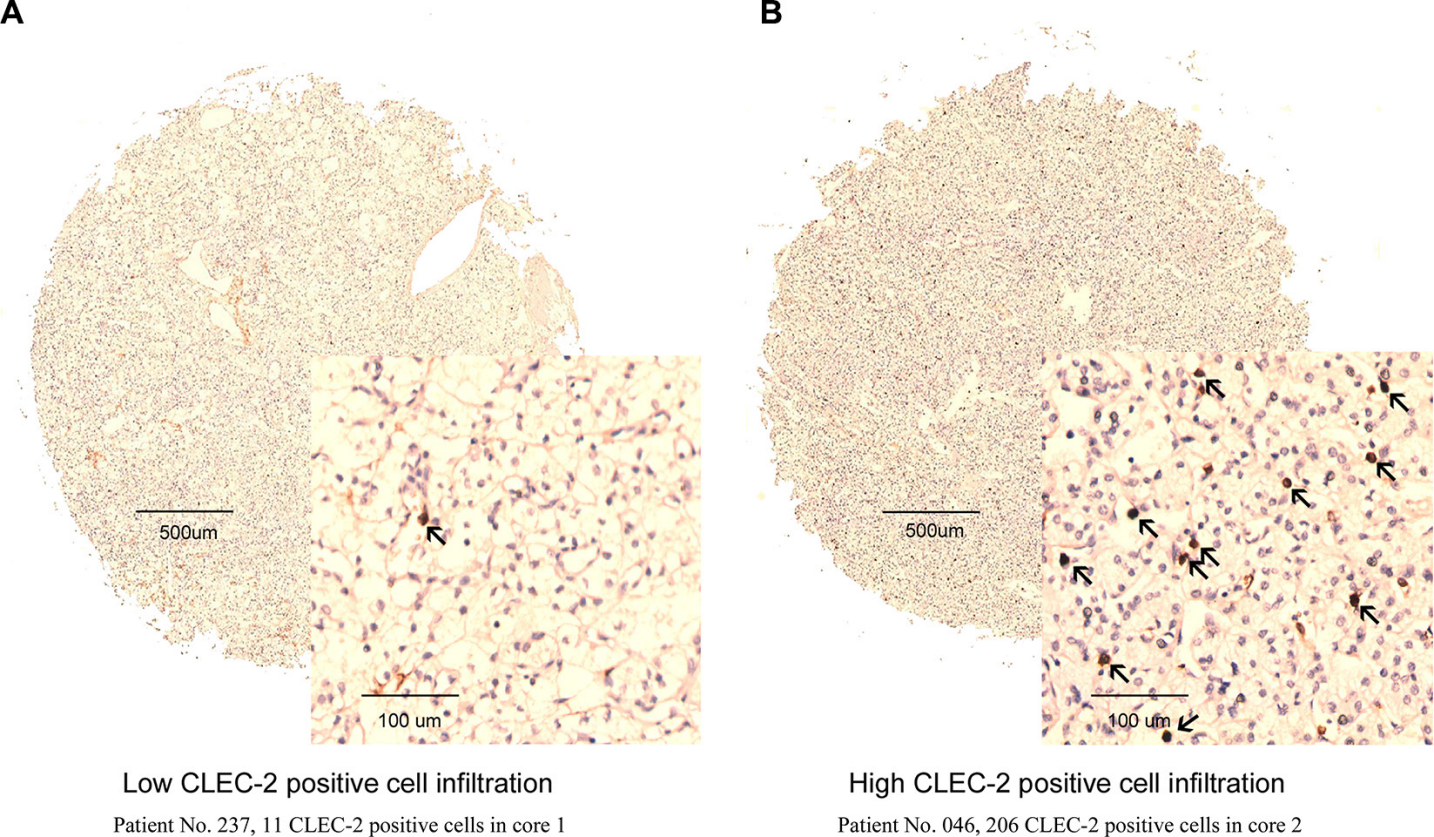
CLEC-2 positive cell infiltration in clear-cell renal cell carcinoma (ccRCC) tissues Representative CLEC-2 immunohistochemical (IHC) images of ccRCC tumor tissues with low CLEC-2 positive cell infiltration (Patient No. 237, 11 CLEC-2 positive cells in core 1) (**A**) and high CLEC-2 positive cell infiltration (Patient No. 046, 206 CLEC-2 positive cells in core 2) (**B**). Arrows indicate CLEC-2 positive cells.

CLEC-2 was mainly expressed in stromal cells compared with tumor tissues (Figure 1A, 1B). A specimen tissue was considered high CLEC-2 positive cell infiltration if it contains more than 53 CLEC-2 positive cells, otherwise low CLEC-2 positive cell infiltration. As we can see from Table S1, CLEC-2 positive cell infiltration is apparently associated with tumor size (*p* = 0.015), pathological T stage (*p* = 0.042), TNM stage (*p* = 0.016) and necrosis (*p* = 0.038). Other clinicopathological parameters of the ccRCC patients were not associated with CLEC-2 positive cell infiltration.

### Association between CLEC-2 positive cell infiltration and clinical outcomes

**Figure 2:**
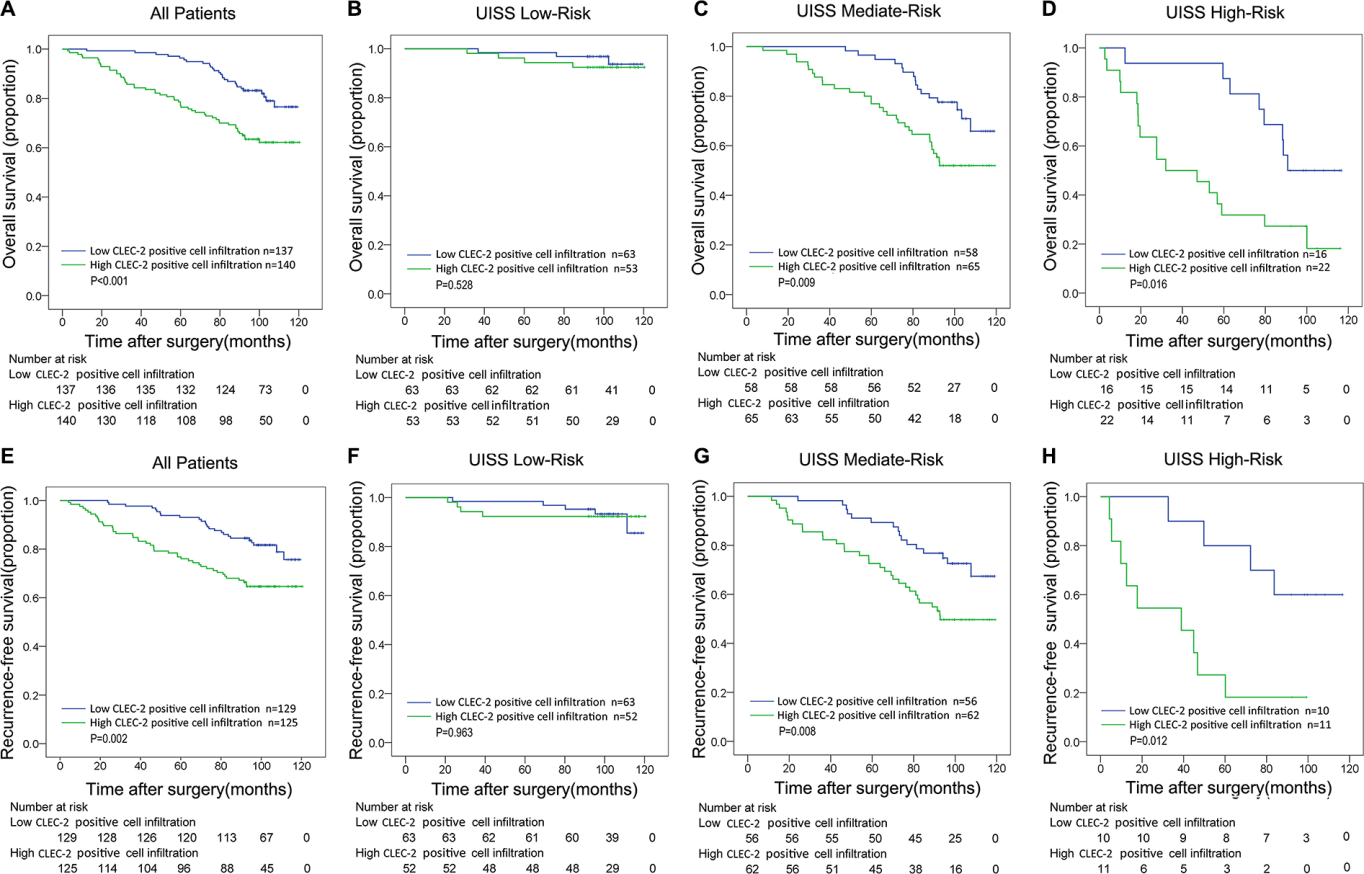
Overall survival (OS) and Recurrence-free survival (RFS) analyses of patients with ccRCC based on CLEC-2 positive cell infiltration Kaplan-Meier analysis of OS in All Patients group (*n* = 277) (**A**); and in UISS Low-Risk group (*n* = 116) (**B**) in UISS Mediate-Risk group (*n* = 67) (**C**) and in UISS High-Risk group (*n* = 74) (**D**); Kaplan-Meier analysis of RFS in All Patients group (*n* = 254) (**E**); in UISS Low-Risk group (*n* = 115) (**F**) in UISS Mediate-Risk group (*n* = 118) (**G**) and in UISS High-Risk group (*n* = 21) (**H**). *P* value was calculated by log-rank test.

The median follow-up time for all available patients was 98.63 months (range 2.63–120.47). The mean follow-up time was 91.06 months. 79 in 277 patients (28.5%) died during the follow up and 68 in 254 patients (26.8%) experienced disease relapse. We compared overall survival and recurrence-free survival according to CLEC-2 positive cell infiltration in order to further investigate the prognostic value of CLEC-2 positive cell infiltration. Obviously, ccRCC patients with high CLEC-2 positive cell infiltration had a poorer OS and RFS compared to those with low CLEC-2 positive cell infiltration. Kaplan–Meier survival analysis was used and as was shown in Figure 2A and 2E, high CLEC-2 positive cell infiltration was a significant negative prognostic predictor for patients included in the study (OS, *p* < 0.001; RFS, *p* = 0.002).

We first conducted univariate analyses using number of CLEC-2 positive cells as a continuous variable. It was a risk factor for OS and RFS (OS, *p* < 0.001; RFS, *p* = 0.001) (Table S2). CLEC-2 positive cell infiltration as a dichotomous variable was also a risk factor for OS and RFS (OS, *p* = 0.001; RFS, *p* = 0.002) (Table S2), indicating an important impact of CLEC-2 positive cell infiltration on clinical outcome. To evaluate the robustness of the prognostic value of CLEC-2 positive cell infiltration and control for confounders, we performed Cox multivariate regression analyses and found that high CLEC-2 positive cell infiltration was still an independent risk factor for both OS and RFS (OS, *p* = 0.004; RFS, *p* = 0.009) (Table 1), together with other clinicopathological parameters like pathological T stage, Fuhrman grade, Necrosis, ECOG PS and distant metastasis. We also found that CLEC-2 positive cell infiltration could stratify ccRCC patients' survival in the UISS mediate-risk and high-risk groups, in which high CLEC-2 positive cell infiltration turned out to be an independent risk factor in both OS and RFS analyses (OS, *p* = 0.009, RFS, *p* = 0.008 in mediate-risk groups; OS, *p* = 0.016, RFS, *p* = 0.012 in high-risk groups) (Figure 2C and 2G, Figure 2D and 2H), while in the low-risk groups it is not statistically significant (Figure 2B and 2E). This probably indicates that CLEC-2 positive cells may function more in patients with higher pathological T stage, Fuhrman grade and/or ECOG PS.

**Table 1:** Proportional hazard model for overall survival and recurrence free survival prediction

Variables	OS (*n* = 277)		RFS(*n* = 254)	
	HR (95%CI)	*P*-value^†^	HR (95%CI)	*P*-value^†^
Pathological T stage		0.001		<0.001
pT1	Reference		Reference	
pT2	2.468 (1.217–5.004)	0.012	2.144 (0.947–4.855)	0.068
pT3	2.968 (1.732–5.088)	< 0.001	3.181 (1.806–5.602)	< 0.001
pT4	4.116 (1.080–15.681)	0.038	11.160 (3.285–37.913)	< 0.001
Distant metastasis				
Yes *vs* No	2.804 (1.421–5.533)	0.003		
Fuhrman grade		< 0.001		< 0.001
1	Reference		Reference	
2	1.641 (0.585–4.600)	0.346	1.297(0.504–3.339)	0.589
3	3.711 (1.231–11.189)	0.020	3.868 (1.376–10.871)	0.010
4	15.148 (3.117–73.613)	0.001	13.530 (2.909–62.929)	0.001
Necrosis				
Present *vs* Absent	1.871 (1.023–3.425)	0.042	1.853(1.010–3.486)	0.046
ECOG PS		0.001		0.001
0	Reference		Reference	
1	2.392 (1.451–3.970)	0.001	2.180 (1.231–3.861)	0.008
2	2.914 (1.079–7.870)	0.035	6.776 (2.547–18.028)	< 0.001
3	4.200 (1.212–14.550)	0.024	5.792 (1.848–18.148)	0.003
CLEC-2 positive cell infiltration				
High *vs* Low	2.065 (1.258–3.390)	0.004	2.057 (1.200–3.524)	0.009

ECOG PS = Eastern Cooperative Oncology Group performance status; HR = hazard ratio; CI = confidence interval; OS = overall survival; RFS = recurrence free survival;

† Data obtained from the Cox proportional hazards model, *P*-value < 0.05 was regarded as statistically significant.

### Extension of current prognostic model with CLEC-2 positive cell infiltration

The sensitivity of the predictive system calculated by C-index could be increased if we combine CLEC-2 positive cell infiltration information into the SSIGN and UISS score system. The SSIGN/UISS simplified three risk groups have been used in this procedure. CLEC-2 positive cell infiltration together with SSIGN score had increased predictive power compared to SSIGN score alone in both OS (C-index 0.744 vs. 0.725) and RFS (C-index 0.672 vs. 0.631) The situation was the same for UISS score, as adding CLEC-2 positive cell infiltration information into the UISS model could also improve its predictive power for both OS (C-index 0.763 vs. 0.743) and RFS (C-index 0.682 vs. 0.638) prediction (Table 2).

**Table 2:** Comparison of the predictive accuracy of the prognostic models

Models	Overall survival		Recurrence free survival	
	C-index	AIC	C-index	AIC
CLEC-2	0.615	845.59	0.615	600.31
TNM	0.706	811.82	0.608	605.91
TNM + CLEC-2	0.738	804.29	0.664	597.78
SSIGN	0.725	809.12	0.631	603.6
SSIGN + CLEC-2	0.744	801.30	0.672	595.93
UISS	0.743	804.84	0.638	608.98
UISS + CLEC-2	0.763	794.91	0.682	600.00
Nomogram	0.813	758.85	0.716	521.21

C-index, concordance index; AIC, Akaike information criterion; SSIGN, Mayo clinic stage, size, grade, and necrosis score; UISS, UCLA Integrated Staging System. C-index and AIC were calculated from 1000 bootstrap sample.

### Prognostic nomograms for OS and RFS

**Figure 3:**
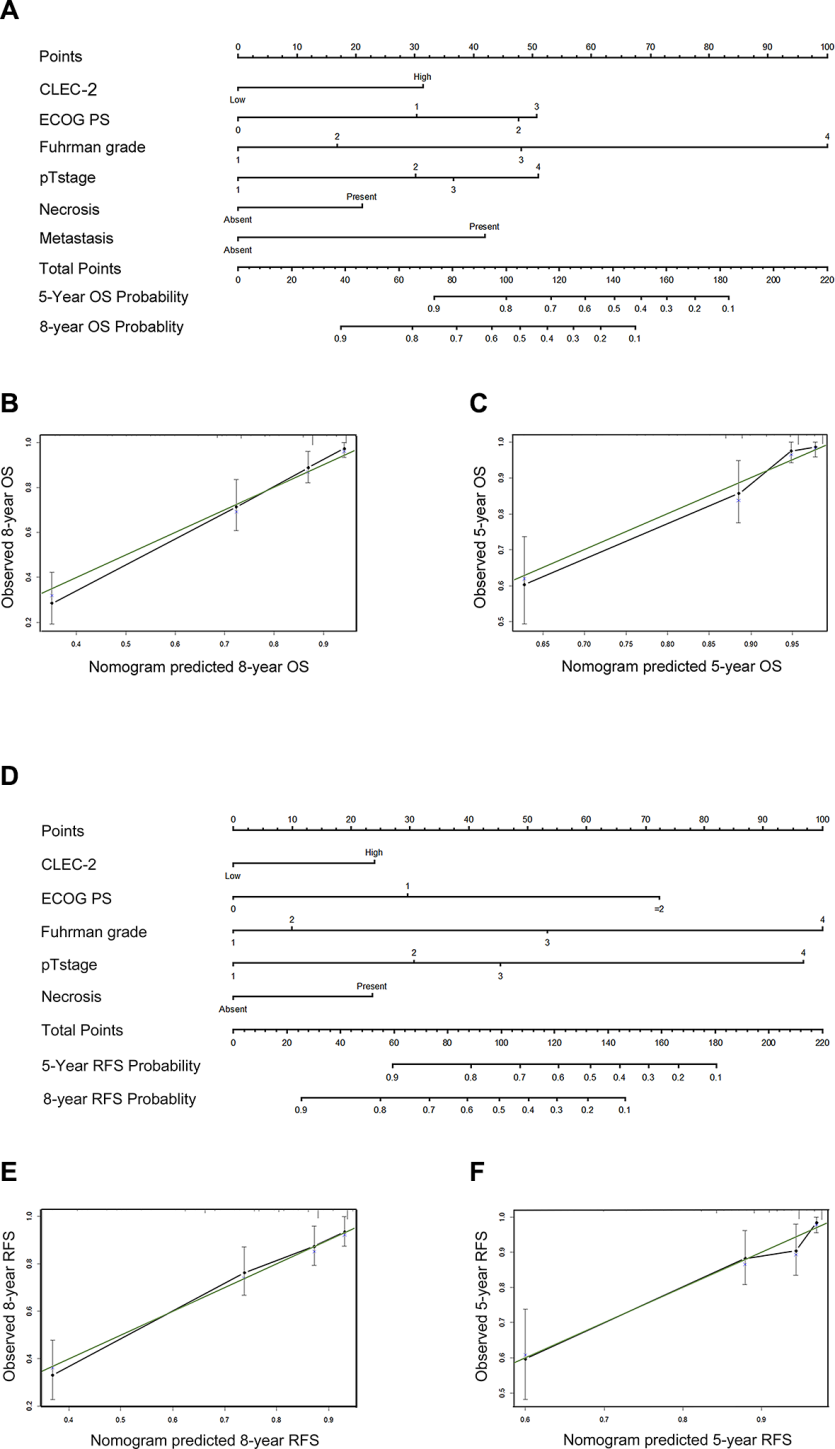
Prognostic nomograms and calibration plots for OS and RFS prediction (**A**) Six independent prognostic factors including CLEC-2 positive cell infiltration, ECOG PS, Fuhrman grade, pathological T stage, necrosis and metastasis were identified and entered into the nomogram. (**B**) Calibration curves for predicting 8-year OS of ccRCC patients. (**C**) Calibration curves for predicting 5-year OS of ccRCC patients. (**D**) Five independent prognostic factors including CLEC-2 positive cell infiltration, ECOG PS, Fuhrman grade, pathological T stage and necrosis were identified and entered into the nomogram. (**E**) Calibration curves for predicting 8-year RFS of ccRCC patients. (**F**) Calibration curves for predicting 5-year RFS of ccRCC patients.

After incorporating significant prognostic factors concluded from Cox multivariate analyses, we established two nomograms to predict 5-year and 8-year ccRCC patients' OS and RFS (Figure 3). Pathological T stage, distant metastasis, Fuhrman grade, necrosis status, ECOG PS and CLEC-2 positive cell infiltration were included. A score was assigned to each level of the variables, and the total score could be used to predict the probability of survival. After performing Bootstrap validations we found that the calibration plots showed good consistency between the predicted and actual observation (Figure 3B, 3C; Figure 3E, 3F). The C-index indicated a good predictive accuracy for nomograms in both OS and RFS (OS, C-index 0.813; RFS, C-index 0.716).

## DISCUSSION

In this study, we detected the infiltration of CLEC-2 positive cell in ccRCC using 277 specimen tissues with immunohistochemistry, and it turned out that high CLEC-2 positive cell infiltration in tumor tissue is correlated with a poor prognosis. As an independent poor prognostic factor for OS and RFS of ccRCC patients, the infiltration level of CLEC-2 positive cells can be added to current prognostic models like TNM stage, UISS and SSIGN in order to improve the predictive accuracy. Moreover, we constructed two nomograms incorporating CLEC-2 positive cell infiltration with other significant parameters derived from multivariate analysis to predict patients' OS and RFS. C-indexes indicated that the two nomograms performed better than current prognostic models.

In this study, the CLEC-2 positive staining was mainly found in tumor stromal cells and it is known that CLEC-2 is mainly expressed on myeloid cells. Some myeloid cells like tumor associated macrophages (TAMs), neutrophils and myeloid-derived suppressor cells (MDSC) could help tumor angiogenesis, invasion, metastasis, and meditate certain immune-suppressive function under specific circumstances. This indicated that those CLEC-2 positive cells in this study might have a role inimmunomodulation and help cancer immunologic escape [6, 9]. According to previous studies, CLEC-2 positive cells could mediate tumor progression mainly in two ways, suppressive immunoregulation and platelet activation. Diego Mourão-Sa et al. found that CLEC-2 could signal via Syk, Ca21 and NFAT, leading to Syk phosphorylation, calcium signaling and NFAT activation in myeloid cells. Notably, by activating NFAT, CLEC-2 can modulate the effect of signals induced by other innate receptors such as toll-like receptors, resulting in selectively increased production of cytokine IL-10 [6]. The strong immune suppressive effects of IL-10 might indicate a role for CLEC-2 positive myeloid cells in immune suppression and tumor progression [10]. Different from other CLRs, receptor crosslinking by anti-CLEC-2 mAb was unable to activate CARD9/NF-κB pathway pathway, which is an important downstream signal of CLRs in inducing pro-inflammatory response [11, 12]. Thus CLEC-2 activation cannot increase serum levels of some pro-inflammatory cytokines like TNF, IL-6, IL1b, IL-1a or IL-12/IL-23p40, which might further prove its immune suppressive role. Therefore, ligation of CLEC-2 might promote cancer progression by creating an immunosuppressive environment and help cancer cell escape from immune surveillance.

On the other hand, CLEC-2 ligation with podoplanin could elicit strong platelets activation [7], which protects them from shear stress and NK cells in the blood stream and serves for tumor cell nestling [13]. Activated platelets release growth factors, promoting tumor angiogenesis and growth. The endogenous ligand of CLEC-2, podoplanin, could increase tumor cells motility by remodeling actin in the cytoskeleton and correlated with the onset of epithelial to mensenchymal transition (EMT), a key role in tumor metastasis [14, 15]. Interaction between podoplanin and CLEC-2 may regulate tumor invasion and metastasis and might be a potential target for therapy of metastasis.

In conclusion, we have identified that CLEC-2 positive cell infiltration correlates with ccRCC patients' survival and can be used as a novel prognostic factor in predicting patients OS and RFS. However, there are still some limitations. This is a retrospective study in nature and the number of patients enrolled is limited. Besides, CLEC-2 may be expressed in varying amounts in different areas of the tumor and the way we chose cutoff point with X-tile might lead to potential overfitting bias. A mutli-centered prospective external validation is needed. Moreover, further experimental studies are also required to identify the detailed role of CLEC-2 positive cells in ccRCC.

## MATERIALS AND METHODS

### Patient selection

The study included a total of 277 patients who received radical or partial nephrectomy due to clear cell RCC in Zhongshan Hospital, Fudan University from Jan 2005 to Jun 2007. All the patients were consecutively included if they met the criterion of having pathologically proven ccRCC, having received partial or radical nephrectomy and possessing available Formalin Fixed Paraffin Embedded (FFPE) specimen of tumor mass (≥ 1 cm^3^). Patients were excluded if they had other malignant tumor before, or histories of adjuvant or neo-adjuvant therapies including targeted therapies. Samples with over 80% necrotic or hemorrhagic area and patients with bilateral tumors were also excluded. Fudan University, Zhongshan Hospital research medical ethics committee approved this study and informed consent was given for the use of clinical specimens in this study.

### Data collection

The primary outcome was OS, which was calculated from the time of operation to the time of death. RFS was defined as the time from nephrectomy to the time of first recurrence. During the first five years, the interval of follow up was three months and one year later then. Data were censored when the patient died or was alive at Jan 30, 2015, the last follow up time. The analysis of recurrence-free survival excluded fifteen patients with metastasis at surgery and eight patients with missing recurrence state.

Baseline clinical characteristics and complete follow-up outcome included in the database were re-examined. Two pathologists (Yuan J. and Jun H.) reviewed the H&E slides to reconfirm histological subtype, stage, and Fuhrman grade. One urologist reassessed all the MRI and CT scans. Histological subtype of ccRCC were reconfirmed according to 2014 EAU guidelines [16]. TNM stage and Fuhrman grade were based on the 2010 AJCC TNM classification and 2012 ISUP consensus, respectively [17, 18]. Patient risks are stratified according to the SSIGN, UISS and SSIGN localized (Leibovich) score according to original scoring algorithm [19–21].

### Immunochemistry

Tissue microarrays were constructed as previously described [22]. This was a different cohort but we used the same method. Immunohistochemical staining was performed on tissue microarrays and primary antibodies against human CLEC-2 (Anti-CLEC-2 antibody, orbr3344, Biobirt, diluted 1/100) was used. Antibody specificity was confirmed by immunochemistry and western blot. The staining results were scanned by a microscopy system (Leica DM6000 B, Leica Microsystems GmbH, Mannheim, Germany). We recorded images with Leica CV-M2CL camera and analyzed them with Leica Ariol 4.0 software. Specimens were considered as high CLEC-2 positive cell infiltration if there were over 53 CLEC-2 positive cells in a tissue core, otherwise low CLEC-2 positive cell infiltration. Each patient had two tissue cores, and the number of CLEC-2 positive cells calculated as the average of them. The cutoff point was selected according to optimal *p* value with X-tile, version 3.6.1 (Yale University, New Haven, Connecticut) [23].

### Statistical analysis

The relationship between CLEC-2 positive cell infiltration and clinicopathological parameters of the patients was assessed by χ^2^ test, Fisher's exact method and Cochran-Mantel-Haenszel χ^2^ test. Survival curves were established with Kaplan-Meier method and compared with log-rank test. Univariate and multivariate Cox proportional hazard models were used to evaluate the HR (Hazard ratio) and 95% CI (confidence interval). Two nomograms were formed to predict the OS and RFS. We calculated concordance index to compare the prognostic or predictive accuracy of different models. Statistical analyses were performed using SPSS Statistics 21.0 (SPSS Inc., Chicago, IL), R software version 3.0.2 with the “rms” package (R Foundation for Statistical Computing, Vienna, Austria) and Stata (version 12.1; StataCorp LP, TX, USA). All statistical tests were 2-sided and *P* < .05 was regarded as statistically significant.

## SUPPLEMENTARY MATERIALS TABLES


