# Supplementary material for: High CLEC-2 expression associates with unfavorable postoperative prognosis of patients with clear cell renal cell carcinoma
Source: Oncotarget. 2016 Aug 25;7(39):63661–8. doi: 10.18632/oncotarget.11606 (PMC5325393; doi:10.18632/oncotarget.11606)
Supplement: Supplementary file 1 [file oncotarget-07-63661-s001.pdf]

High CLEC-2 expression associates with unfavorable postoperative prognosis of patients with clear cell renal cell carcinoma

Supplementary Materials

Table S1: Clinical characteristics of patients according to CLEC-2 positive cell infiltration

Characteristics	Patients		CLEC-2 positive cell infiltration		
	n	%	low	high	*P*-value
All patients	277	100	137	140	
Age, years					0.875*
mean ± SD	55.71 ± 12.66		55.83 ± 13.04	55.59 ± 12.31	
Gender					0.519†
Female	86	31	40	46	
Male	191	69	97	94	
Tumor size, cm					0.015*
mean ± SD	4.71 ± 2.56		4.34 ± 2.06	5.08 ± 2.92	
Pathological T stage					0.042‡
pT1	173	62.5	96	77	
pT2	27	9.7	7	20	
pT3	73	26.4	33	40	
pT4	4	1.4	1	3	
Pathological N stage					0.988†
pN0	275	99.3	136	139	
pN1	2	0.7	1	1	
Distant metastasis					0.109†
No	262	94.6	133	129	
Yes	15	5.4	4	11	
TNM stage					0.016‡
I	168	60.6	94	74	
II	23	8.3	6	17	
III	67	24.2	32	35	
IV	19	6.9	5	14	
Fuhrman grade					0.203‡
1	30	10.8	16	14	
2	203	73.3	99	104	
3	41	14.8	19	22	
4	3	1.1	3	0	
Necrosis					0.038†
Absent	241	87.0	125	116	
Present	36	13.0	12	24	
ECOG PS					0.505†
0	200	72.2	100	100	
1	62	22.4	31	31	
2	11	4.0	5	6	
3	4	1.4	1	3	
UISS category					0.143‡
low risk	115	41.5	62	53	
mediate risk	124	44.8	59	65	
high risk	38	13.7	16	22	
SSIGN category					0.153‡
0–3	215	77.6	110	105	
4–7	56	22.2	26	30	
8+	6	2.2	1	5	

**t*-test for continuous variables, †χ^2 test or Fisher's exact test, ‡Cochran-Mantel-Haenszel χ^2 test, *P*-value < 0.05 was regarded as statistically significant; ECOG PS=Eastern Cooperative Oncology Group performance status.

Table S2: Univariate analyses of characteristics associated with overall survival and recurrence free survival

Variables	OS (*n* = 277)			RFS (*n* = 254)		
	**Hazard Ratio**	**95%CI**	***P*-value†**	**Hazard Ratio**	**95%CI**	***P*-value†**
Age, years	1.031	1.012–1.050	< 0.001	1.205	1.006–1.046	0.011
Gender			0.953			0.956
Male *vs* Female	0.953	0.590–1.593		0.956	0.594–1.636	
Tumor size	1.195	1.110–1.288	< 0.001	1.195	1.101–1.298	< 0.001
Pathological T stage			< 0.001			< 0.001
pT1	1.000	Reference		1.000	Reference	
pT2	3.526	1.837–6.767	< 0.001	3.506	1.705–7.210	0.001
pT3	3.269	1.992–5.366	< 0.001	2.923	1.720–1.720	< 0.001
pT4	7.923	2.404–26.113	0.001	14.854	5.110–43.178	< 0.001
N classification						
N1 *vs* N0	1.336	0.175–10.189	0.780			
Distant metastasis						
Yes *vs* No	6.322	3.412–11.716	< 0.001			
TNM stage			<0.001			<0.001
I	1.000	Reference		1.000	Reference	
II	3.317	1.589–6.885	< 0.001	3.755	1.871–7.536	< 0.001
III	3.360	1.968–5.736	< 0.001	2.729	1.588–4.690	< 0.001
IV	10.791	5.695–20.446	< 0.001	14.914	7.044–49.419	< 0.001
Fuhrman grade			< 0.001			< 0.001
1	1.000	Reference		1.000	Reference	
2	1.913	0.689–5.306	0.213	1.344	0.531–3.405	0.533
3	6.156	2.132–17.779	0.001	4.762	1.784–12.708	0.002
4	7.941	1.775–35.520	0.007	6.336	1.511–26.563	0.012
Necrosis						
Present *vs* Absent	2.709	1.629–4.504	< 0.001	3.006	1.754–5.152	< 0.001
ECOG PS			< 0.001			< 0.001
0	1.000	Reference		1.000	Reference	
1	3.307	2.064–5.299	< 0.001	2.767	1.642–4.664	< 0.001
2	3.999	1.689–9.471	0.002	4.703	1.845–11.988	0.001
3	5.370	1.652–17.456	0.005	8.234	2.907–23.325	< 0.001
CLEC-2 positive cells	1.005	1.002–1.007	< 0.001	1.004	1.002–1.007	0.001
CLEC-2 positive cell infiltration			0.001			0.002
High *vs* Low	2.278	1.430–3.627		2.154	1.318–3.520	

ECOG PS= Eastern Cooperative Oncology Group performance status; CI=confidence interval; OS= overall survival; RFS = recurrence free survival; †Data obtained from the Cox proportional hazards model, *P*-value < 0.05 was regarded as statistically significant.
